# The presence of a lipoma in the Eustachian tube: a case report

**DOI:** 10.1186/1752-1947-5-436

**Published:** 2011-09-06

**Authors:** Zhuofu Liu, Dehui Wang, Quan Liu

**Affiliations:** 1 The Department of ENT, Eye, Ear, Nose and Throat Hospital of Fudan University, Shanghai, 200032, China

## Abstract

**Introduction:**

The incidence of lipoma in the Eustachian tube is very rare, and to the best of our knowledge, has not been reported in the literature. Tumors that form in the cartilaginous portion of the Eustachian tube can be successfully removed by an endoscopic approach.

**Case presentation:**

We report an incidentally-detected lipoma of the Eustachian tube in a 34-year-old Asian woman with a six-year history of persistent otitis media in her right ear. Our patient underwent surgery five years ago for the possibility of a choanal polyp, but her ear symptoms continued to be problematic following the surgery. Our patient was examined at our hospital, and computed tomography revealed a well-defined, hypodense, non-enhancing lesion involving the right Eustachian tube, measuring 1.6 × 2.4 cm. The mass was excised using an endoscopic approach, and was found to originate from the cartilaginous portion of the Eustachian tube. The tumor was sent for histopathologic evaluation. The postoperative course went smoothly, and our patient recovered during follow-up over the next five months.

**Conclusion:**

Lipoma of the Eustachian tube is very rare compared with other tumors. Improved radiologic modalities aid the diagnosis of this benign tumor. Endoscopic removal of the tumor is possible and has helped in early recovery.

## Introduction

Lipomas are benign tumors and present as painless soft tissue masses commonly seen in adults [1]. They can arise from many parts of the body, but are most commonly found in the subcutaneous tissue of the neck and trunk. According to their location, they are also categorized as subcutaneous, submucous or intramuscular lipomas. Apart from fat cells, lipomas may also contain other tissue components, such as fibrous, nervous or vascular tissue [2]. Lipomas originating from the Eustachian tube are extremely rare. Herein we report a case of a lipoma arising from the Eustachian tube.

### Case presentation

**Figure 1:**
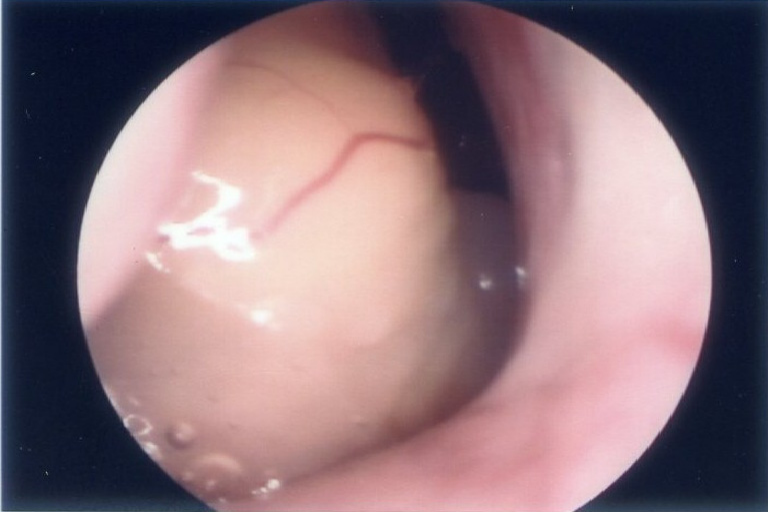
**Endoscopic examination showed a well-encapsulated, yellow-colored tumor in the right nasopharynx**.

**Figure 2:**
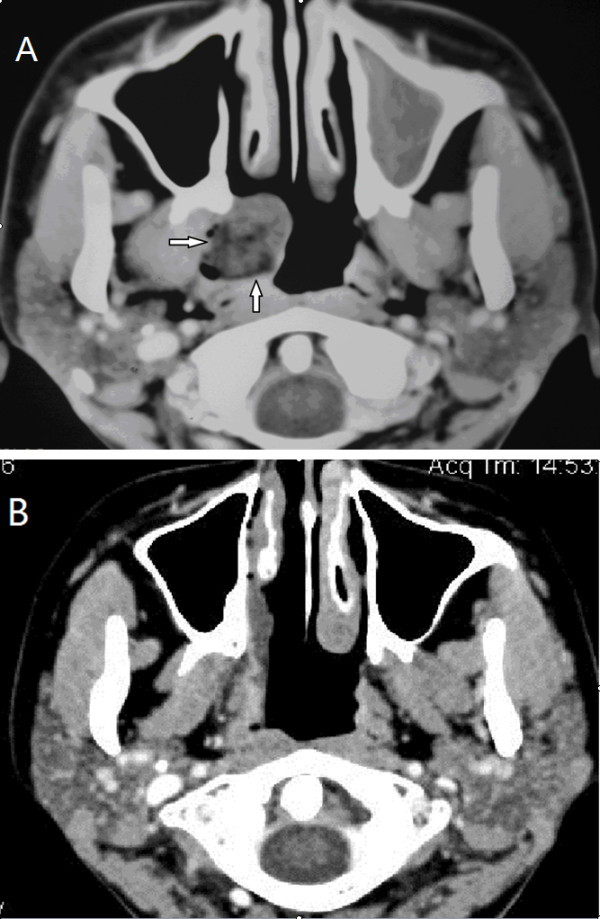
**(A) Axial section of a contrast-enhanced CT scan shows the hypodense, non-enhancing lesion (arrow) in the right Eustachian tube**. (B) Five months after surgery, axial section of the contrast-enhanced CT scan at the same level shows no residual tumor.

A 34-year-old Asian woman presented at our hospital, with a six-year history of persistent right otitis media with effusion. In addition, on presentation, our patient was suffering from tinnitus and a sensation of 'fullness' in her right ear. Our patient underwent unilateral myringotomy through the insertion of a pressure equalization tube, which was kept in place up until a few months ago because the ear symptoms continued to cause our patient problems. Five years previously our patient was operated on at her local hospital for a neoplasm in the right nasopharynx due to a possible choanal polyp; the histological results of the neoplasm could not be obtained. On this occasion, our patient presented to our hospital with right nasal obstruction. Endoscopic examination displayed a well-encapsulated, yellow-colored tumor in the right side of her nasopharynx (Figure 1A). Contrast-enhanced computed tomography (CT) was done, which showed a well-defined, hypodense (relative to the surrounding muscle), non-enhancing lesion involving the right Eustachian tube, measuring 1.6 × 2.4 cm (Figure 2A). Examination of our patient revealed a tympanic membrane perforation on the right side, and a T-tube was present in the right tympanic membrane.

**Figure 3:**
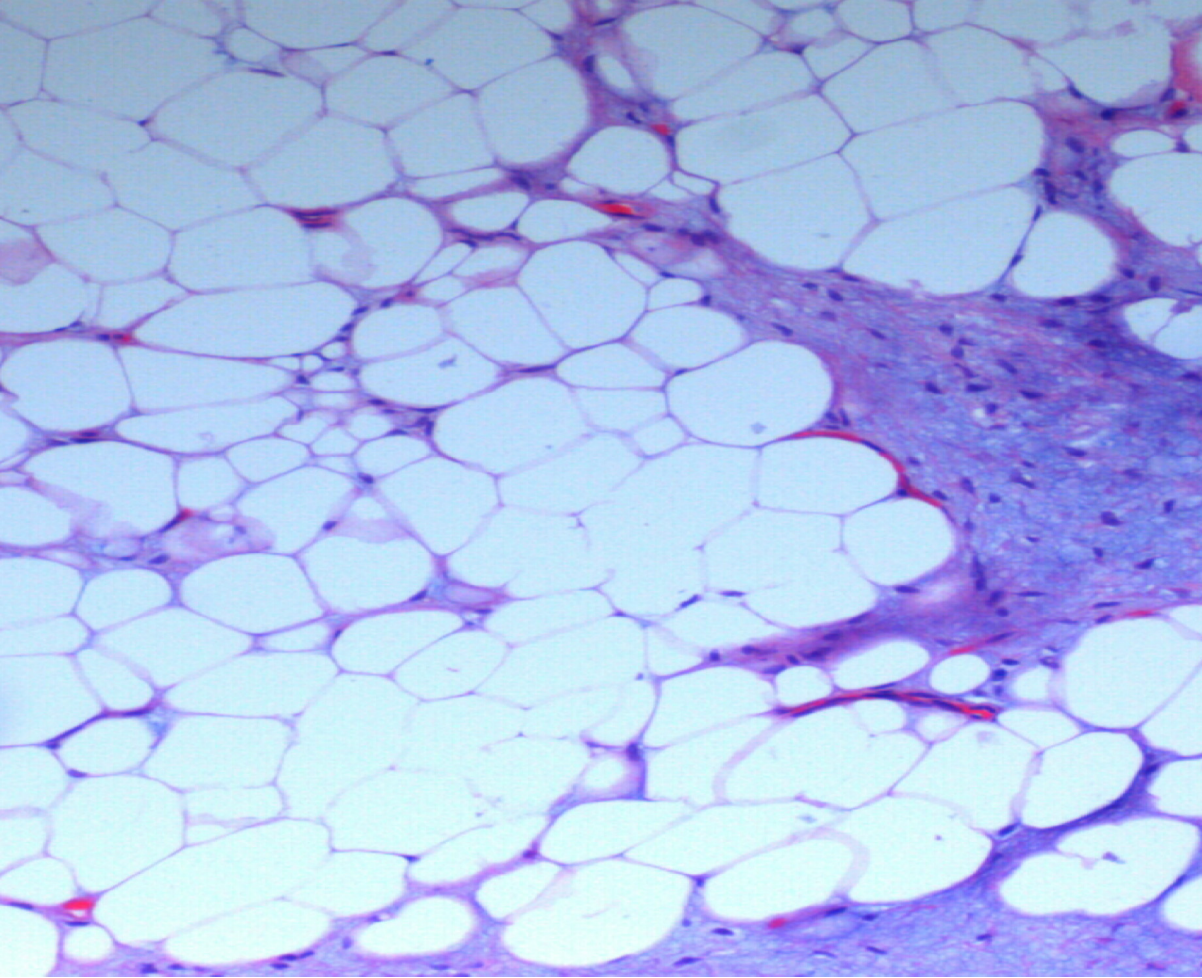
**High power of the specimen shows a lipomatous lesion with some fibroblastic tissue (H&E, × 200)**.

The mass was excised under general anesthesia by endoscopy. Good exposure of the tumor was obtained by removal of part of her middle turbinate, and the tumor was subsequently removed completely. The tumor was found to arise from the cartilaginous portion of the Eustachian tube. The excised specimen showed a well-encapsulated, yellow-colored tumor, with a soft consistency and had the gross appearance of adipose tissue. Histological examination confirmed the diagnosis of fibrolipoma (Figure 3). Our patient was discharged the day after surgery and received antibiotics for three days.

Two months after this surgery, endoscopic examination showed the nasopharynx with no sign of recurrence of the tumor. The tympanic membrane perforation on the right was healing. Five months later, a contrast-enhanced CT scan showed no residual tumor (Figure 2B), and our patient had no tinnitus and no sensation of 'fullness' in her ear.

## Discussion

Lipomas are the most common benign tumors, and are derived from the mesenchyme. They are composed of mature adipose tissue, and several subtypes occur when other mesenchymal elements are present [3], for example fibrous tissue, nervous tissue or vascular tissue. Histologically, lipomas can be classified as conventional lipoma, fibrolipoma, angiolipoma, spindle cell/pleomorphic lipoma, myxolipoma, chondroid lipoma, osteolipoma or myolipoma [1]. Lipomas can be singular or multiple; small or of variable size; and symptomatic or asymptomatic. The majority of these benign tumors are asymptomatic, and symptoms that do arise are usually due to pressure effects on adjacent structures.

The typical clinical presentation of tumors involving the Eustachian tube consists of chronic ear drainage with recurrent episodes of otitis media. This is caused by the obstruction of the Eustachian tube by the tumor. Upper airway obstruction is a result of extension of the tumor into the nasopharynx.

Tumors of the Eustachian tube may arise primarily from within, or secondarily by invading it from surrounding structures. These lesions include, amongst others, tumors of dermoid cysts [4], teratoma [5], malignant mucosal melanoma [6] and synovial sarcoma [7], Here, to the best of our knowledge, we report the first case of a lipoma originating from the Eustachian tube. We found four cases of lipomas which were found to arise from the middle ear [3,8-10], two of them invading the Eustachian tube, and even extending to the nasopharynx. Lipomas have been characterized as showing homogeneous fatty attenuation upon CT imaging, and show different intensities according to the different components they contain.

The surgical approach involving the Eustachian tube is a difficult procedure due to the regional anatomy. Zollner [11] suggested a good approach to the tube via an open mastoid cavity, or at least the tympanic entrance of the bony part of the tube, which is used by many aural surgeons [3,4,9]. In 1969, House [12] designed the middle fossa approach for Eustachian tuboplasty. However, this procedure is not widely accepted. With the advent of transnasal endoscopic surgery, more and more tumors located in the nasopharynx and lower part of the Eustachian tube have been treated in this way. Lin [7] removed a synovial sarcoma from the lower portion of the anterior cushion of the Eustachian tube by transnasal endoscopic surgery. For this case, an endoscopic approach was the best choice considering the tumor was contained within the cartilaginous portion of the Eustachian tube and nasopharynx.

## Conclusion

To the best of our knowledge, this is the first report of a lipoma originating from the cartilaginous portion of the Eustachian tube, and the tumor was removed successfully by an endoscopic approach.

## Consent

Written informed consent was obtained from the patient for publication of this case report and any accompanying images. A copy of the written consent is available for review by the Editor-in-Chief of this journal.

## Competing interests

The authors declare that they have no competing interests.

## Authors' contributions

ZL was the major contributor in writing the manuscript. DW performed the surgery and helped in preparation of the final manuscript. QL did the endoscopic examination and postoperative follow-up of our patient. All authors read and approved the final manuscript.
